# The role of virtual reality in breast cancer survivors: A scoping review

**DOI:** 10.1016/j.pmedr.2025.103032

**Published:** 2025-03-14

**Authors:** Zhuyue Ma, Li Sun, Yuanyuan Zhang

**Affiliations:** a Jiangsu Province Hospital of Chinese Medicine & Affiliated Hospital of Nanjing University of Chinese Medicine, 210029, China; b Jiangsu Province Hospital of Chinese Medicine & Affiliated Hospital of Nanjing University of Chinese Medicine, 211100, China

## Abstract

**Objective:**

This scoping review aims to analyze features, functions, and effectiveness evaluation of VR in the application of breast cancer care.

**Methods:**

A scoping review was conducted based on the framework suggested by Arksey and O'Malley. Eight electronic databases (i.e., PubMed, EMBASE, Cochrane Library, Web of Science, CINAHL, CNKI, China Wanfang Database, and China Biology Medicine disc.) was searched. Articles published from inception till 01 April 2024.

**Results:**

After screening 16,606 articles, a total of 21 papers were included. 10 immersive VR studies and 8 non-immersive VR studies. The main themes identified included the classification and function of VR, content elements of VR for Breast cancer care (e.g., symptoms, rehabilitation, and education), and evaluation of the effectiveness of VR (e.g., clinical outcomes, experience, adverse effects).

**Conclusion:**

VR in 2 categories has potential applicability in symptom, rehabilitation, and educational management, with high satisfaction and acceptance by most patients. However, the current application of VR in breast cancer is limited, and there is still a need to optimize VR component design and focus on its long-term impact on patients and related stakeholders in the future.

## Highlights

• Virtual Reality apply to symptoms, rehabilitation, and education in Breast cancer survivors.
• Breast cancer survivors have favorable perceptual feedback on Virtual Reality.
• There are adverse reactions to the Virtual Reality use.
• Component compatibility and human-computer interaction in Virtual Reality need to be improved.

## 1 Introduction

According to the latest data from the International Agency Research on Cancer (IARC), Breast cancer (BC) accounted for 2.2 million new cases for the first time, almost 11.7 % of the total cancer incidence, and has become one of the most common cancers worldwide(Dyba et al., 2021). Moreover, new breast cancer patients are predicted to increase by more than 46 % by 2040(Heer et al., 2020). Although the number of new cases has been increasing dramatically in recent years, the 5-year survival rate of BC patients is also remarkable, reaching over 90 %. This is due to positive and promising technological advances in treatment, which unfortunately still produce stressful sequelae. We discovered that self-reporting and thorough management of BC patients frequently reveals issues such as limb swelling, fatigue, pain, anxiety, psychological distress due to body image disruption, radiotherapy-related side effects, dread of rehabilitation exercises, and so on. Sebri et al. showed that cancer patients often complain of the presence of chemo brain, cognitive impairment, and cancer-caused fatigue(Sebri and Pravettoni, 2023). Breast cancer remains as a stigmatized pathology that subjects women to functional losses and relevant social and emotional changes,in addition to significant impact in their lifestyles, which may result in a pronounced negative impact on QL(Garcia et al., 2017).

Most task-oriented rehabilitation exercises and long-term psychosocial interventions are currently applied in BC care, most commonly cognitive-behavioral therapy (CBT) and Mindfulness-Based Stress Reduction (MBSR). Yang et al. showed in a CBT-based systematic review that psychological interventions that promote positive changes in cognition, thinking, and behavior are effective in reducing depression, anxiety, and other negative psychiatric disorders, as well as relieving pain, sleep disorders, and other physical symptoms(Yang et al., 2024). A Rct of breast cancer survivors, MBSR, conducted an eight-week group therapy program that reduced cognitive impairment after receiving chemotherapy through a variety of exercises in positive thinking skills, including mindfulness of breath, thoughts, bodily sensations, sounds, and everyday activities, with weekly 2.5-h sessions and one full retreat day(Duval et al., 2022). Some studies have shown that exercises following exercise prescriptions not only contribute to the functional rehabilitation of the affected limb after surgery, but also improve the physiological health of patients, possibly by increasing blood perfusion, activating muscle-cancer cell pathways, and ultimately by improving fatigue, muscle strength, and aerobic work capacity(Delrieu et al., 2020). They were instructed in problem-solving and relaxation techniques, were more persistent and cooperative throughout the session, and rarely withdrew abruptly. This may be related to the reduction of the stress response by processing negative emotions through neurological and biological factors. However, these traditional methods are time-consuming, cumbersome, and costly, and their effectiveness often depends on the patient's ability to absorb and understand. Traditional rehabilitation therapies are repetitive and mechanical, and patients tend to lose interest in the process and have difficulty maintaining it, which affects the training effect. In particular, poor adherence to rehabilitation training has been a difficult problem to address. Therefore, it is critical to improve recovery training tolerance and maintain mental health levels. There is an urgent need to incorporate novel, innovative, and practical approaches to support BC care.

In healthcare, VR has the potential to improve the nursing care quality by productivity and efficiency of healthcare at a lower cost. First proposed in the 1960s, the concept of VR is an artificial intelligence technology based on multidisciplinary cooperation(Cipresso et al., 2018). It uses the computer as the core architecture to calculate and simulate a three-dimensional (3D) virtual environment and combines it with other somatosensory devices (e.g., a somatosensory handle, sensing helmet, and 3D glasses). VR systems are divided into two categories: immersive and non-immersive, and the difference between the two depends on the degree to which the user is isolated from the physical environment while interacting with the virtual environment, further determining the user's sense of presence in VR(Chirico et al., 2016). Immersive VR is defined as the user being placed in an artificial world, wearing a head-mounted device and headphones, without external access to light and sound, which projects images with corresponding sound and manipulates a computer mouse or handle to get a sense of touch, providing a dynamic sense of presence that allows the users to change their current behavior as they go, i.e., immersive and interactive multi-sensory stimulation. Another type of non-immersive VR is characterized that the user can join the virtual world created in front of the computer screen, but still communicate with the real world(Chirico et al., 2016). Several reviews have investigated the applicability and efficiency of VR in healthcare and found that VR can improve functional exercise, pain, anxiety, cognitive impairment, etc. in different groups. Gomez's study showed that training physical function brought positive outcomes and provided the ability to perform daily life tasks. Given that VR is essentially an interactive user technology, Găină's study conducted applications that distracted pain perception with significant efficacy(Găină et al., 2022); Rutkowski's study also found that improving chemotherapy tolerance through distraction is a promising strategy(Rutkowski et al., 2021). In addition, VR systems provide both cognitive training and affective relief via custom integrative rehabilitation games. Whereas BC management is multi-faceted burden management, VR has limited specificity in providing quality nursing care.

Several systematic reviews have assessed the effectiveness of symptom and rehabilitation management of VR, lacking a clinical care systematic review and not detailing their impact on the quality of care(Zhang et al., 2022). Likewise, Zasadzka's systematic review focused only on assessing the effectiveness of VR as an effective tool for BC treatment, lacking subjective patient evaluations and the challenges encountered during the intervention(Zasadzka et al., 2021). Therefore, a scoping review of the use and effectiveness of VR in BC care is necessary. The purpose of this scoping review of the literature is to determine: 1) Summarize what areas of VR technology are used in the care of breast cancer patients? 2) Types of VR equipment? 3) How do BC patients perceive it during VR application? 4) What are the adverse effects?

## 2 Methods

A scoping review approach is appropriate as it aims to map the available evidence on a given topic to clarify broad review questions(Munn et al., 2018). The review was conducted following the PRISMA-SCR for scoping reviews guidelines Appendix 1(Tricco et al., 2018), employing a six-step process: formulating the research question, identifying and selecting relevant studies, study selection, data graphing, synthesizing the results, and translating the results(Matamala-Gomez et al., 2022). The research was registered on the OSF.

### 2.1 Search strategy

The search strategy and search terms were co-designed by two researchers for iterative searches in PubMed, and CNKI to identify the most accurate index terms. Articles published from inception to April 1, 2024, were systematically searched in eight databases: PubMed, EMBASE, Cochrane Library, Web of Science, CINAHL, CNKI, China Wanfang Database, and China Biology Medicine Disc. Reference lists of all included articles were also searched. Search strings for the database are shown in Appendix 2.

### 2.2 Study selection

To be included in the scoping review, research related to measuring or focusing on VR in BC patients. The inclusion criteria for the study were (1) patients diagnosed with breast cancer; (2) physical、psychological and cognitive outcomes as the focus of observation; (3) a description of VR use; and (4) discussed the patients' perceptions of VR. Qualitative, quantitative, or mixed-methods studies are included to assess the impact of first-hand relevant evidence on VR. Gray literature such as conference papers, unpublished papers, research protocols only, and literature not published in English or Chinese were excluded(Tricco et al., 2018). Eligibility criteria were set a posteriori to ensure familiarity with the current literature on the topic and to avoid missing relevant articles.

### 2.3 Data selection and extraction

Initial identification and screening of titles and abstracts were independently performed by two authors against predefined inclusion and exclusion criteria.After removing duplicates, two authors independently screened titles and abstracts and then reviewed the full text. Both authors then assessed the full text against the same criteria. Conflicts between reviewers were settled by the third author. The data graph template (Appendix 3) was drafted by the lead author with input from the review team and then piloted by two randomized investigators through five studies. No disagreements arose between the two researchers, and a standardized data extraction form was ultimately established, increasing the reproducibility of the study, which included (country, setting, study design, aims, outcomes, VR device, replaced senses, intervention time, adverse effects, etc.). Final decisions on inclusion were made by consensus.

### 2.4 Data analysis and presentation

We adopted a combination of narrative synthesis and tables for each review question for data analysis and presentation. Narrative synthesis could summarize and compare similarities and differences among extensive research findings on a specific topic, whereas tabulation could present details of research results.

## 3 Results

### 3.1 Study selection and study characteristics

**Fig. 1:**
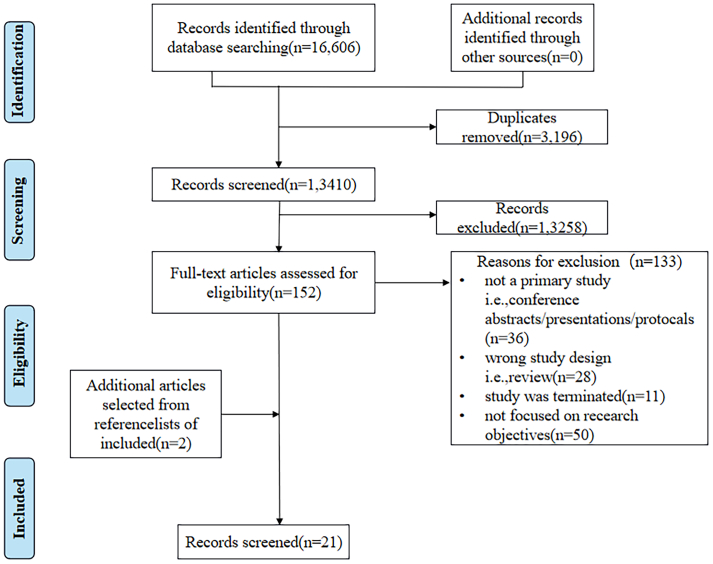
Search sequence.

The initial search strategy yielded 16,606 articles. After the first stage of screening to remove duplicate articles and filter titles and abstracts, 152 papers were filtered. In the second stage, after full-text reading and evaluation, 19 papers were accepted for inclusion. In the third stage, references to included studies were reviewed and 2 applicable papers were included. In total, 21 papers were further analyzed in Fig. 1(Schneider et al., 2003, Schneider et al., 2004, Harless et al., 2009, McGarvey et al., 2010, House et al., 2016, Aixiang et al., 2018a, Aixiang et al., 2018b, Jimenez et al., 2018a, Jimenez et al., 2018b, Xiaomin et al., 2019, Liumei et al., 2019, Bani Mohammad and Ahmad, 2019, Atef et al., 2020, Chirico et al., 2020, Feyzioğlu et al., 2020, Haiyan et al., 2021, Liu et al., 2021, Buche et al., 2021, Zhou et al., 2021, Basha et al., 2022, Reynolds et al., 2022). Out of the 21 articles included, the majority were developmental studies conducted in a medical facility (ward or outpatient or rehabilitation center) (*n* = 10, 47.6 %)(Schneider et al., 2003, Schneider et al., 2004, McGarvey et al., 2010, Jimenez et al., 2018a, Jimenez et al., 2018b, Bani Mohammad and Ahmad, 2019, Atef et al., 2020, Feyzioğlu et al., 2020, Buche et al., 2021, Basha et al., 2022). 2 articles were conducted in the laboratory(Chirico et al., 2016, Chirico et al., 2020). One each in a community(House et al., 2016) and home intervention(Reynolds et al., 2022), and 6 articles were not detailed. 11 articles conducted RCT validation, 5 pilot studies, 3 mixed studies and 2 crossover design studies. Surprisingly, the use of VR in clinical BC care was diverse, with 8 articles for anxiety relief, 6 for physical rehabilitation, 6 for cognitive rehabilitation, and 5 for pain relief, among others. In addition, in conjunction with the study design of the VR intervention, we categorized the 21 included articles and found that the rct studies with the strongest evidence were characterized by a prospective approach, adequate sample size, and methodological rigor, and that the intervention outcomes were primarily pain, anxiety, and recovery indicators. The study characteristics are detailed in Table 1. And Study characteristics summary is shown in Table 2. The clinical outcomes of the different study designs are shown in Table 3.

**Table 1:** Characteristics of included studies (including authors, country, study design, objectives, intervention group, control group, outcome instruments and results)

Author & year published	Country	Study setting	Study design	Aim	Treatment Status	Intervention group	Control group	Outcomes/Tools	Results
Atef D 2020	Egypt	Physiotherapy center	RCT	reduce lymphedema	after surgery	N-IVR	PNF	EAV DASH-9	Both VR and PNF help to reduce lymphedema after mastectomy, and the effects of both techniques are similar.
Bani Mohammad E 2019	Jordan	General wards	RCT	reduce pain and anxiety	after surgery	IVR	Morphine	VAS SAI MMSE	Immersive virtual reality technology is an effective distraction intervention for treating pain and anxiety in breast cancer patients.
Basha M A 2022	Egypt	Outpatient clinics	RCT	reduce lymphedema	after surgery	N-IVR	RT	VAS DASH-9 ROM SF-36	Xbox Kinect showed higher significant improvements in pain intensity and shoulder ROM, which improved patients' activities of daily living and quality of life, and inspired more social and activity interactions.
Buche H 2021	France	Physiotherapy center	four-arm RCT	Promote recovery	after surgery	IVR	Contemplative VR/Music therapy/Classical scar massage session	ITC–SOPI SAM SAI QC	showed an increase in positive emotions (i.e., joy and happiness) and a decrease in anxiety regardless which support methods were offered. Participatory VR created a feeling of more intense spatial presence.
Chirico A 2020	Italy	Laboratory	three-arm RCT	reduces anxiety and improves emotional state	chemotherapy	IVR	MT/UC	SAI POMS VRSQ	both VR and MT are useful interventions for alleviating anxiety and for improving mood states in breast cancer patients during chemotherapy. Moreover, VR seems more effective than MT in relieving anxiety, depression, and fatigue.
Feyzioğlu Ö 2020	Turkey	General wards	RCT	Promote recovery	after surgery	N-IVR(KBRG)	SPTG	VAS ROM muscle strength DASH TKS	Both groups detected significant changes in pain, ROM, muscle strength, grip strength, functionality, and TKS scores after the treatment
Hareless WG2009	USA	Not reported	Mixed study	assess the effectiveness of virtual dialogues	after surgery	N-IVR(Virtual Dialogue)		Technological feasibility Acceptance Knowledge gain	Virtual dialogue is feasible and acceptable
House G 2016	USA	Community	pilot study	Pain relief and Promote recovery	after surgery	N-IVR	N-IVR	NRS UE range of motion UE strength and functional assessments Cognitive and emotive outcomes	Outcomes indicate improvement in cognition, shoulder range, strength, function and depression.
Jimenez Y A 2018a	Australia	General wards	pilot study	Knowledge teaching and anxiety reduction	radiotherapy	N-IVR(VERT)	CG	RT knowledge STAI RT experience	reports the high value of VERT breast cancer-targeted education programs in improving RT knowledge and perhaps decreasing patient anxiety.
Jimenez Y A 2018b	Australia	General wards	Mixed study	report on the patient evaluation of the newly developed education program for breast cancer patients	radiotherapy	N-IVR(VERT)		Education Program Evaluation Patient Perception of VERT Images	The evaluation of the program content and delivery was highly rated, with notable acknowledgement to 3D visual features of the VERT system.
McGarvey E L 2010	USA	Breast center	Mixed study	the effects of the HAAIR system; reduce alopecia-related distress	chemotherapy	N-IVR(The HAAIR Intervention)	SCG	BSI-18 IHQ Cope	the HAAIR program is a patient-endorsed educational and supportive complement to care for women facing chemotherapy-related alopecia
Reynolds LM2022	Australia	Home	pilot study	Relief of physical and mental symptoms	after surgery	IVR		EQ-5D-5L FACIT-Fatigue BPI DASS-SF	VR promotes physical and mental health
Schneider S M 2003	USA	Outpatient clinics	Crossover design	Reduce chemotherapy-related symptom distress	chemotherapy	IVR		MMSE PFS SAI SDS	VR alleviates anxiety
Schneider S M 2004	USA	Outpatient clinics	Crossover design	Reduce chemotherapy-related symptom distress	chemotherapy	IVR		PFS SAI SDS	VR alleviates symptom distress
Zhou Z 2021	China	Laboratory	pilot study	Promote recovery	after surgery	IVR	UC	SUS PQ SSQ	VR promotes functional rehabilitation of the upper extremity
Jin A X2018	China	Not reported	RCT	Promote recovery	after surgery	IVR	UC	SF-36	VR platform facilitates physical and mental recovery after BC surgery
Jin A X2018	China	Not reported	RCT	Promote recovery	after surgery	IVR	UC	ROM Adherence to rehabilitation training Limb edema measurement	VR helps patients recover limb function
Chen X M2019	China	Not reported	pilot study	Helps restore cognition and improve daily living skills	chemotherapy	IVR(pre)	VR(post)	MoCA ADL	VR improves cognitive function and enhances daily living skills
Zhu L M2019	China	Not reported	RCT	Promote recovery	after surgery	?	UC	ROM Adherence to rehabilitation training Complications	VR promotes physical rehabilitation
Lin H Y2021	China	Not reported	RCT	Promote recovery	after surgery	?	UC	ROM Adherence to rehabilitation training ADL FACT-B MoCA	VR helps restore cognitive function and improves life skills
Yang L2021	China	Not reported	RCT	Helps restore cognition	chemotherapy	?	UC	MoCA SF-36 SUPPH Adherence to rehabilitation training	VR improves cognitive function and quality of life

EAV:excess arm volume; DASH:Disability of the Arm, Shoulder, and Hand (DASH) questionnaire; PNF:proprioceptive neuromuscular facilitation; VAS:visual analog scale; SAI:The State Anxiety Inventory; MMSE:Mini-Mental State Examination; SF-36:The QoL was assessed by the Medical Outcomes Study Short-Form; ROM:range of motion; ITC–SOPI:The Independent Television Commission–Sense of Presence Inventory; SAM:Self-Asssessment Manikin; QC:*questionnaire on cybersickness; RT:Resistance training; MT:*Music therapy; POMS:the short version of Profile of Mood States; VRSQ:The Virtual Reality Symptom Questionnaire; KBRG:the Kinect-based rehabilitation group; SPTG:the standardized physical therapy group; VERT:Virtual Environment for Radiotherapy Training; CG:control group; SCG:Standardized Care group; BSI-18:The Brief Symptom Inventory,with 18 items; IHQ:The Importance of Hair Questionnaire; Cope:The Brief Cope; EQ-5D-5L:the five-level EuroQol five-dimensional questionnaire; FACIT-Fatigue:The Functional Assessment of Chronic Illness Therapy Fatigue scale; BPI:The Brief Pain Inventory; DASS-SF:The 21-item short version of the Depression, Anxiety, and Stress Scales; PFS:Revised Piper Fatigue Scale; SDS:Symptom Distress Scale; SUS:the System Usability Scale; PQ:the Presence Questionnaire scale; SSQ:the Simulator Sickness Questionnaire; MoCA:the Montreal Cognitive Assessment; ADL:Activity of Daily Living; FACT-B:functional assessment of cancer therapy-breast; SUPPH:Strategies Used by People to Promote Health; UC: usual care.

**Table 2:** Study characteristics summary.

Study characteristics	N	(%)
Study design		
RCT	11	52,4
pilot study	5	23.8
Crossover design	2	9.5
Mixed study	3	14.3
Countyr of origin		
Egypt	2	9.5
Arab	1	4.8
France	1	4.8
Italy	1	4.8
Istanbul	1	4.8
USA	5	23.8
Australia	3	14.3
China	7	33.3
Settings		
General wards (ie. breast center)	4	19.0
Outpatient clinics	3	14.3
Physiotherapy center	2	9.5
Laboratory	2	9.5
Community	1	4.8
Home	1	4.8
Treatment Status		
after surgery	13	61.9
chemotherapy	6	28.6
radiotherapy	2	9.5
VR use cases for clinical nursing		
Fatigue	3	14.3
Lymphoedema	4	19.0
Pain	5	23.8
Hair loss	1	4.8
Anxiety	8	38.1
Depression	2	9.5
Exercise fear	1	4.8
Limb function	6	28.6
Cognitive function	6	28.6
Muscle strength	2	9.5
Knowledge	2	9.5
Adherence to rehabilitation training	4	19.0

**Table 3:** Clinical outcomes of VR interventions with different study designs.

Study design	Author&year published	Outcomes/Tools	Results
RCT	Atef D 2020	EAV DASH-9	Both VR and PNF help to reduce lymphedema after mastectomy, and the effects of both tecniques are similar.
	Bani Mohammad E 2019	VAS SAI MMSE	Immersive virtual reality technology is an effective distraction intervention for treating pain and anxiety in breast cancer patients.
	Basha M A 2022	VAS DASH-9 ROM SF-36	Xbox Kinect showed higher significant improvements in pain intensity and shoulder ROM, which improved patients' activities of daily living and quality of life, and inspired more social and activity interactions.
	Buche H 2021	ITC–SOPI SAM SAI QC	showed an increase in positive emotions (i.e., joy and happiness) and a decrease in anxiety regardless which support methods were offered. Participatory VR created a feeling of more intense spatial presence.
	Chirico A 2020	SAI POMS VRSQ	both VR and MT are useful interventions for alleviating anxiety and for improving mood states in breast cancer patients during chemotherapy. Moreover, VR seems more effective than MT in relieving anxiety, depression, and fatigue.
	Feyzioğlu Ö 2020	VAS ROM muscle strength DASH TKS	Both groups detected significant changes in pain, ROM, muscle strength, grip strength, functionality, and TKS scores after the treatment
	Jin A X2018	SF-36	VR platform facilitates physical and mental recovery after BC surgery
	Jin A X2018	ROM Adherence to rehabilitation training Limb edema measurement	VR helps patients recover limb function
	Zhu L M2019	ROM Adherence to rehabilitation training Complications	VR promotes physical rehabilitation
	Lin H Y2021	ROM Adherence to rehabilitation training ADL FACT-B MoCA	VR helps restore cognitive function and improves life skills
	Yang L2021	MoCA SF-36 SUPPH Adherence to rehabilitation training	VR improves cognitive function and quality of life
Pilot study	House G 2016	NRS UE range of motion UE strength and functional assessments Cognitive and emotive outcomes	Outcomes indicate improvement in cognition, shoulder range, strength, function and depression.
	Jimenez Y A 2018a	RT knowledge STAI RT experience	reports the high value of VERT breast cancer-targeted education programs in improving RT knowledge and perhaps decreasing patient anxiety.
	Reynolds LM2022	EQ-5D-5L FACIT-Fatigue BPI DASS-SF	VR promotes physical and mental health
	Zhou Z 2021	SUS PQ SSQ	VR promotes functional rehabilitation of the upper extremity
	Chen X M2019	MoCA ADL	VR improves cognitive function and enhances daily living skills
Mixed study	Jimenez Y A 2018b	Education Program Evaluation Patient Perception of VERT Images	The evaluation of the program content and delivery was highly rated, with notable acknowledgement to 3D visual features of the VERT system.
	McGarvey E L 2010	BSI-18 IHQ Cope	the HAAIR program is a patient-endorsed educational and supportive complement to care for women facing chemotherapy-related alopecia
	Hareless WG2009	Technological feasibility Acceptance Knowledge gain	Virtual dialogue is feasible and acceptable
Crossover design	Schneider S M 2003	MMSE PFS SAI SDS	VR alleviates anxiety
	Schneider S M 2004	PFS SAI SDS	VR alleviates symptom distress

### 3.2 VR applied to BC clinical care cases

Five intervenable somatic symptoms and two common psychological symptoms were identified: fatigue(*n* = 3)(Schneider et al., 2003, Chirico, Maiorano et al. 2020, Reynolds et al., 2022), lymphoedema(*n* = 4)(Aixiang J, Xiaomin C et al. 2018, Liumei Z, Jiajia Y et al. 2019, Atef et al., 2020, Basha et al., 2022), cognitive disorders(*n* = 6)(Schneider et al., 2003, Schneider, Prince-Paul et al. 2004, Xiaomin C 2019, Bani Mohammad and Ahmad, 2019, Haiyan L, Lihua W et al. 2021, Liu Y, Xiaomei W et al. 2021, Reynolds et al., 2022), pain(*n* = 5)(House et al., 2016, Bani Mohammad and Ahmad, 2019, Feyzioğlu et al., 2020, Haiyan L, Lihua W et al. 2021, Basha et al., 2022), hair loss(*n* = 1)(McGarvey et al., 2010), anxiety(*n* = 8)(Schneider et al., 2003, Schneider, Prince-Paul et al. 2004, Jimenez, Cumming et al. 2018, Jimenez, Wang et al. 2018, Bani Mohammad and Ahmad, 2019, Chirico, Maiorano et al. 2020, Buche et al., 2021, Reynolds et al., 2022) and depression(*n* = 2)(Chirico, Maiorano et al. 2020, Reynolds et al., 2022). In addition, the utility of VR for rehabilitation management can be presented by the following indicators. The DASH was used to assess disorders and measure disability of the upper limb and monitor change and function over time(*n* = 3)(Atef et al., 2020; Feyzioğlu et al., 2020; Basha et al., 2022). ROM indicated the mobility of the shoulder joint in internal rotation, abduction, forward flexion, posterior extension, external rotation, and internal retraction, as measured by digital goniometer in the study(*n* = 5)(Aixiang J, Xiaomin C et al. 2018, Liumei Z, Jiajia Y et al. 2019, Feyzioğlu et al., 2020, Haiyan L, Lihua W et al. 2021, Basha et al., 2022), hand-held dynamometer assesses muscle strength in shoulder flexion, abduction, and external rotation by maximum isometric muscle contraction, dynamometer handle to measure grip strength(*n* = 2)(Feyzioğlu et al., 2020, Basha et al., 2022). All VR application effects showed statistically significant differences. Besides, whether patients completed each exercise component regularly and quantitatively according to the rehabilitation training program was set as compliance, as mentioned(*n* = 4), but unspecific compliance data could not be verified. Specifically, we were told that the VR intervention enhanced the knowledge and experience of BC patients(*n* = 2)(Harless et al., 2009, Jimenez, Cumming et al. 2018), which also suggests that VR can be used as a novel health education tool by healthcare professionals.

### 3.3 VR technology apply

Of the included studies, 9 used immersive VR(IVR), and 9 used non-immersive VR(N-IVR). IVRs typically include devices that can interact with the virtual environment, such as head-mounted displays, motion tracking systems, headsets, and joystick-style controllers. The patient wore an image that can be projected with corresponding sounds and manipulated by a computer mouse to change the image(Schneider et al., 2003). Each patient was invited to use a specific joystick to shape her environment (e.g., control the weather, plant trees or flowers, spawn animals.)(Buche et al., 2021). Each patient has a controller to interact with the virtual environment, such as walking through the forest, observing different animals, climbing mountains and swimming in the sea, etc.(Chirico, Maiorano et al. 2020). Patients can experience 360° VR nature scenes, such as:a beach where the participant can write words in the sand or the sky; a waterfall where the participant can stack stones; and a mountain range where the participant can jump between different locations among mountaintops and lakes. The patient-controlled the handle position and tracks the coordinates of the upper limb activity information, allowing the calculation of the upper limb activity trajectory(Zhou et al., 2021). The intervention uses mostly visual-auditory and tactile, and sometimes tactile and olfactory feedback, a process of total immersion in the virtual environment created. N-IVR typically consists of a computer screen-based that can join the virtual world, be replaced by a visual, and still be able to interact with the external world. A series of exercise routines can be practiced through video games(Atef et al., 2020). The Xbox 360 Kinect™ does not require a game controller, since human motion through 3D depth sensors, transferring relevant data to the computer system without physical contact(Feyzioğlu et al., 2020; Basha et al., 2022), The laboratory robotics platform completes training tasks by adjusting the gravity load on the upper limbs in combination with some virtual games(House et al., 2016). The radiotherapy training virtual environment provided clinical equipment that simulates radiotherapy, including linear gas pedals and treatment beds, both with realistic motion and sound, displaying radiation fields, internal patient anatomy and RT target areas, and other visualization options for educational training(Jimenez, Cumming et al. 2018). Psychoeducation on hair loss using the computerized imaging system HAAIR (Help with Adjustment to Alopecia Image Recovery)(McGarvey et al., 2010). Based on a computer program that allows users to communicate verbally and eye-to-eye with virtual experts, asking questions that can be answered directly(Harless et al., 2009). Specific VR device use cases and features are displayed in Table 4.

**Table 4:** Characteristics of VR programs.

Author(s), year	VR equipment	Immersion level	Replaced senses	How to Intervene/Intervention design elements	Intervention time(each/total)	Intervention side effects
Atef D 2020	Nintendo Wii® Video Games	N-IVR	Vision	Complete tennis, triceps extension and rhythm boxing by freely moving the shoulder joint	30 min/4 week	
Bani Mohammad E 2019	CD-ROM a headmounted display with headphones	IVR	Vision Aural	Wearing a headset to select one of the 2 scenarios offered by the CD	Time to peak effect after VR exposure to painkillers	
Basha M A 2022	Xbox Kinect (3D body camera)	N-IVR	Vision Aural	Upper body rehabilitation exercises through Xbox Kinect games (darts, bowling, boxing, ping pong, fruit ninja and beach volleyball)	/8 week	
Buche H 2021	Oculus Go ®Head-mounted displays	IVR	Vision Aural kinaesthetic	The experimenter helped the participants put on the VR headset and program the desired environment for direct access to relaxation.	10 min/10 month	Cybersickness (4)
Chirico A 2020	head-mounted glasses (Vuzix Wrap 1200 VR)	IVR	Vision Aural Kinaesthetic smell	Each patient has a controller to interact with the virtual environment, participants explored an island, by walking through a forest, observing different animals, climbing a mountain, and swimming in the sea.	20 min/During chemotherapy	Cybersickness symptoms
Feyzioğlu Ö 2020	Xbox 360 Kinect™	N-IVR	Vision Aural	Play video games	35 min/6w	
Hareless WG2009	the Virtual Conversations® model	N-IVR	Vision Aural	A natural voice processor enhanced by an intelligent prompting system enables users to talk to virtual experts and make direct eye contact.	30 min	
House G 2016	BrightArm Duo	N-IVR	Vision Aural	Play 9 kinds of two-handed games with screen images(a) Breakout 3D, (b) Card Island, (c) Remember that Card, (d) Musical Drums, (e) Xylophone, (f) Pick & Place, (g) Arm Slalom, (h) Avalanche and (i) Treasure Hunt.	20-50 min/8 week	Lymphoedema
Jimenez Y A 2018	VERT	N-IVR	Vision kinaesthetic	Education content: showing description of localisation inside the patient (a), the path of the radiation beam with respect to the planning target volume and organs at risk (b) and the treatment room (c)	1 h	
Jimenez Y A 2018	VERT	N-IVR	Vision kinaesthetic	Education content: showing description of localisation inside the patient (a), the path of the radiation beam with respect to the planning target volume and organs at risk (b) and the treatment room (c)	1 h	distress
McGarvey E L 2010	The HAAIR computer imaging system	N-IVR	Vision	Different wigs and hairstyles to choose from	60-90 min	
Reynolds LM2022	Pico Goblin VR Headphones LCD screen a controller	IVR	Vision Aural Kinaesthetic	The three experiences are as follows: 1) a beach where the participant can write words in the sand or the sky; 2) a waterfall where the participant can stack stones; and 3) a mountain range where the participant can jump between different locations among mountaintops and lakes.	10 min/1 week	Claustrophobic dizzy/nauseous
Schneider S M 2003	Sony PC Glasstron PLM-S700	IVR	Vision Aural Kinaesthetic	Participants wore the head-mounted device during their intravenous chemotherapy treatment. Participants chose from three CD-ROM based scenarios; (Oceans Below®, A World of Art®, or Titanic: Adventure Out of Time®).	several hours	
Schneider S M 2004	Sony PC Glasstron PLM-S700	IVR	Vision Aural Kinaesthetic	Participants wore the head-mounted device during their intravenous chemotherapy treatment. Participants chose from three CD-ROM based scenarios; (Oceans Below®, A World of Art®, or Titanic: Adventure Out of Time®).	several hours	
Zhou Z 2021	an HTC VIVE Pro2.0 device (including 2 handle controllers, 2 infrared base stations and 1 HMDs device), and the helmet is connected to a computer (Dell Ravener G15 laptop, 3060 graphics card, Intel Core i7 processor, Windows 10 operating system).	IVR	Vision Aural Kinaesthetic	rehabilitation exercise modules and puzzle game modules		
Jin A X2018	VR somatosensory equipment (helmets, joysticks, data gloves)	IVR	Vision Aural Kinaesthetic	Fun video games and soft music	30 min/3 month	
Jin A X2018	VR somatosensory equipment (helmets, joysticks, data gloves)	IVR	Vision Aural Kinaesthetic	Fun video games and soft music	15–30 min/3 month	
Chen X M2019	Somatosensory controllers and personal computers	N-IVR	Vision Aural Kinaesthetic	video games	8 week	
Zhu L M2019	Rehabilitation Training Virtual Reality System	?	Vision	Stage-based rehabilitation	15–30 min/3 month	
Lin H Y2021	Rehabilitation Training Virtual Reality System	?	Vision	Stage-based rehabilitation	8 week	
Yang L2021	Personal computers and physical interaction equipment	?	Vision Aural Kinaesthetic	Virtual game training	8 week	

Buche's study used the ITC (spatial sense questionnaire) to assess users in four areas: VR devices, degree of involvement in immersive tasks, influence of the external environment, and negative effects caused by the instruments, showed that participatory lacks a certain sense of active interactive navigation, but is more natural(Buche et al., 2021). Zhou Z used PQ (Presence Questionnaire) to assess the immersion and sense of presence used by users to emphasize the characteristics of engagement and immersion in the simulated environment(Zhou et al., 2021). The duration of the intervention varied widely among the 21 included studies, ranging from 1 week to 10 months.

### 3.4 BC patients' perception of VR

Most patients reported being receptive to the VR intervention, with some key findings including that the users thought the headset was easy to use and were highly likely to use VR again. Comments from participants in the VR experience indicate that “With my lack of mobility that's resulted from my illness, I enjoyed the VR as it made me feel like I'm not housebound. I could immerse myself elsewhere and it helped take the focus off my pain.”(Reynolds et al., 2022). Patients in the VERT education program said “Visualizations are extremely helpful”, “Understand the whole process from beginning to end”, and “I am no longer worried and feel very calm”(Jimenez, Cumming et al. 2018). All patients stated that they “enjoyed” or “were glad” that they used the system to demonstrate to themselves or to learn what to happen with hair loss(McGarvey et al., 2010). Moreover, over half (60 %) of the women volunteered suggestions to improve the system or commented on features that they liked or disliked. This also demonstrates that the HAAIR system development has been successful in terms of ease of use and perceived usefulness, and the corresponding feedback received from users who used it. Inclusion of these articles containing qualitative insights in this study appears to be unreported barriers to VR use, citing only the presence of the following adverse effects.

### 3.5 Adverse effects

Five studies indicated adverse reactions, with cybersickness, lymphoedema, distress, claustrophobia, and dizziness/nauseous occurring. QC, VRSQ, and SSQ were used to assess patients' side effects in VR interventions, and we discovered that the measurement of VR disease has not been widely used, while the prevalence of VR disease remains problematic. Notably, before this, we excluded people who prevented the risk of VR-related discomfort, including patients with vestibular disorders, a reported history of motion sickness, a history of epilepsy, or a severe psychiatric disorder. Most studies indicated no other adverse effects.

## 4 Discussion

21 articles were included in this scoping review, and we summarized the scope of VR in the clinical care of BC patients. To our surprise, VR intervention can play a crucial role in symptom management, rehabilitation management, and health education for BC patients. Furthermore, most included studies were RCTs, which provide strong evidence to confirm the utility of VR technology in facilitating BC clinical care. In addition, the devices, functions of VR technology, and patients' perceptions of VR interventions are summarized for the first time in this paper.

This study found the efficacy of VR applications on many symptoms management in BC patients, including somatic symptoms (fatigue, lymphedema, cognitive impairment, pain, hair loss) and psychological symptoms (anxiety and depression). In a review, it was shown that VR intervention effectively reduce patients' anxiety, which in turn affected their fatigue and pain levels. This improvement in well-being also boosts their immune system, and these beneficial effects have been demonstrated in different clinical applications, including chemotherapy, painful procedures, and hospitalization(Żydowicz et al., 2024a, Żydowicz et al., 2024b). VR should be considered as a feasible and acceptable adjunctive therapy for improving patients' quality of life. Bani et al. showed that immersive VR was only utilized during the use of pain medication and the duration of the intervention was short, and in the future, multi-arm RCTs could be conducted comparing VR with other non-pharmacological therapies, such as art therapy and music therapy(Bani Mohammad and Ahmad, 2019). Whereas previous studies have shown that IVR interventions have a similar effect on anxiety and pain relief as traditional sedatives, the most recognized mechanism for reducing pain thresholds and anxiety is attentional distraction. Particularly, feelings coexist between symptoms; for example, patients tend to be filled with anxiety when they perceive pain as well(Wender et al., 2022). This is related to the nature of IVR interventions, which were being in a virtual environment and a high level of interactive feedback endows the user with subjective, rich emotions that become distracting when flooding their senses.

Fatigue occurs in actively treated patients with an incidence of approximately 60–90 %(O'Regan et al., 2019). In the Reynolds et al. study, reducing patient fatigue was the most significant finding during the one-week VR intervention and throughout the three-week study period, and demonstrated more beneficial effects over time(Reynolds et al., 2022). A Meta-analysis showed that VR could be a potential tool to secure better fatigue levels for all patients and highlighted the need for additional longitudinal studies in this area(Ioannou et al., 2020). This may be related to the fact that fatigue is a long-term effect of treatment, even lasting for years. The present study configured the controller to interact with the virtual environment, and patients could subjectively adjust the intensity of training, thus correcting the fatigue state; not only that, patient autonomy helps to increase motivation and compliance with VR therapy, promoting continuous training to improve muscular endurance and reduce exercise fatigue(Qian et al., 2020).

Depression causes many patients to be unable to participate in subsequent effective treatment and is therefore of great concern in symptom management. Whereas the better effect of VR on depressed mood states in BC patients is this study may not only be a function of distraction mechanisms but also the possibility that the VR component carries an increasing sense of gaming experience, which reduced the level of depression in patients. Furthermore, in Reynolds' study, the observed reductions in anxiety did not meet the threshold for a clinically important decrease(Reynolds et al., 2022). One reason for this may be that our sample reported relatively low anxiety from the outset, which may have created a floor effect whereby the intervention failed to reduce anxiety further. Evidence from neuropsychological research found that positive game-playing experiences trigger the release of hormones such as endorphins and striatal dopamine, that are responsible for feelings of pleasure and well-being(Mehrabi et al., 2021). VR in this study reduced the distress of BC survivors with hair loss and provided an effective anticipatory response to the psychosocial distress that precedes female hair loss.

Specifically, lymphedema, a patient-specific complication after mastectomy with removal of axillary lymph nodes, develops in 75 % percent of patients within three years of surgery, and once it occurs, it cannot be reversed and is likely to lead to disability(Greene and Goss, 2018). Only one meta-analysis showed a significant difference in the incidence of BC lymphedema with VR interventions. This is groundbreaking for VR interventions and proposes that future studies with larger samples are needed to confirm their preventive effects. Besides, lymphedema is also the cause of many patients' complaints of upper extremity dysfunction, so upgrading lymphedema function is closely relevant to rehabilitation management. Recently, augmented reality methods (AR) incorporates features such as an accurate combination of the digital and physical world and the interactions made between them in real time. AR, such as the three-dimensional laser scanner (3DLS), have emerged as promising tools for measuring upper limb lymphedema. The 3DLS performs a surface scan of the upper limb, creating a precise 3D model. It projects a laser dot onto the upper limb, and the sensor measures the distance to the surface(Hameeteman et al., 2016). This technology is cost-effective, user-friendly, reproducible, and extremely precise.

This study found significant advantages of VR in BC rehabilitation management, which mainly includes rehabilitation of physical and cognitive functions. Research on VR for rehabilitation management has focused more on patients with neurological disorders, and studies have shown that VR has been incorporated into neurological rehabilitation programs to improve functional mobility, balance, gait, and cognitive performance of the upper extremities in patients with neurological disorders(Jin et al., 2022). VR-based rehabilitation systems (e.g. Xbox 360 Kinect game, BrightArm Duo rehabilitation system) have been repeatedly proven to enhance handgrip strength, muscle strength, etc.(House et al., 2016; Feyzioğlu et al., 2020). Consultations and follow-up visits in a personalized virtual environment maintain a high level of interaction and engagement between patients and healthcare providers, providing an engaging rehabilitation environment(Żydowicz, Skokowski et al. 2024). Further, the neurorehabilitation capacity of VR interventions may be related to the optimization and enhancement of brain compensatory mechanisms, immersion enhances the activation of motor brain regions and directly trains the central nervous system; cognitive functions are also trained multidimensionally through different scenarios of the virtual environment with ecological implications. For rehabilitation exercise adherence, the 2 included Chinese papers did not indicate specific adherence values, suggesting the potential ability of data feedback exchange with VR technology. In also, to analyze the differences in patient adherence and intervention effects brought about by different VR components, participatory VR does not seem to bring more cognitive stimulation and positive effects than immersive VR, which is a shortcoming of the three-arm or multi-arm experiments in terms of study design.

The application of VR to radiotherapy virtual environment education in this study was valid, and several meta-analyses showed that the group of VR technology in education was mainly healthcare, and was demonstrated by the learning outcomes(Choi et al., 2022). The mechanism of action of this innovative educational tool may have its roots in psychology, where patients' beliefs, fears, and expectations influence their experiences before and after treatment, and where the knowledge gained from immersion techniques leads to clearer knowledge and easier patient cooperation. It is worth noting that the virtual educational environment needs to take into account not only the technical details of the treatment process but also all possible stimuli that may influence the patient's response. Virtual rehabilitation systems are thought to rely solely on visual and auditory stimuli, possibly due to the mechanical and boring nature of the repetitive video content and the lack of controllers that can be interacted with, resulting in patients feeling more like they are watching an immersive documentary(House et al., 2016). In contrast, the use of joystick controllers adds stimuli for haptic interaction and multisensory engagement, such as planting trees, walking in a forest, and writing on a beach(Feyzioğlu et al., 2020; Buche et al., 2021), in line with our expectations of how objects and people react in the situations described, thus generating behavior in the virtual environment.

The VR intervention for BC patients in this study had a high level of immersion, i.e., patients could easily develop a sense of immersive presence. The virtual rehabilitation systems are considered to rely solely on visual and auditory stimuli, which may be due to the mechanical and boring nature of the repetitive video content and the lack of controllers that can interact with it, resulting in patients feeling more like they are watching an immersive documentary(House et al., 2016). In contrast, the use of joystick controllers adds tactile interaction and multi-sensory engagement stimuli such as planting trees, walking in the forest, writing on the beach(Buche et al., 2021; Reynolds et al., 2022), in line with our expectations of how objects and people will react in the described situation, thus generating behavior in the virtual environment. Yet, many studies lack a subjective and objective evaluation of the patient's spatial sense, and there are potential technological advances in the gap between multisensory sensory-generated stimuli. Surprisingly, some patients in the study suggested creating more virtual environment scenarios, and these suggestions reinforced the importance of user involvement in developing VR designs that fully consider patients' preferences and needs. This review also incorporates qualitative studies that provide insight into the patient experience to further evaluate and improve VR user interfaces and interactions to optimize personalized use of VR in the future.

## 5 Strengths and limitations

It seems that presenting results in a scoping review is often a challenge for reviewers, so tables are useful for linking concepts related to the review questions in order to categorize or analyze them. Hence, it was an essential decision to prioritize the setting of tables for data standardization in the methodology section of this study. We conducted a VR scoping review of the field of breast cancer care that was innovative and database-registered, utilizing a framework based on Arksey and O'Malley that is better suited to descriptively mapping the literature on emerging topics, yielding high-quality evidence-based practice outcomes(Khalil et al., 2021).

Although this scoping review provides a comprehensive review of the scope of VR technology in BC care, there are several limitations. First, only English and Chinese literature from specialized databases was included, which has potential publication bias and reduces the comprehensiveness of the review. Second, no attempt was made to assess the quality of the different study designs, focusing on generalizations of scope. Furthermore, to analyze the differences in patient compliance and intervention effects brought about by different VR components, participatory VR does not seem to bring more cognitive stimulation than immersive VR, which is a shortcoming of the lack of three-armed or multi-armed experiments in the study design. Finally, according to step 6 of the framework, stakeholders and healthcare professionals were not consulted on the preliminary results of this review, and follow-up may be needed to develop the potential for VR applications on multi-subjects.

## 6 Conclusions and future directions

VR has gained popularity for its potential to improve the delivery and quality of healthcare services by optimizing the care process. This scoping review takes an integrated view of patient perceptions of the use of this technology through the application of existing VR technologies in BC care, highlighting the applicability and potential of VR to improve the quality of care.

Future, it is necessary to integrate multidisciplinary perspectives, such as digital intelligence to strengthen human-machine linkage; ergonomics to strengthen VR component compatibility; sociology to strengthen ethical norms, etc., as well as in-depth discussions on the long-term role and cost implications of VR. With VR as an emerging technology, its economic toxicity but the potential cost burden that comes with it cannot be ignored. In future, financial navigation in oncology in managing finances, optimizing the use of health insurance, and applying for policy financial assistance programs could be moderately introduced to innovative technologies such as VR. Future VR studies in which adverse effects are generated should be counted in detail and their impact on intervention adherence or effectiveness should be explored. Based on this, optimizing hardware and software systems to overcome accessibility and ergonomic challenges to boost comfort in VR use, such as the addition of adjustable font sizes and color correction tools assistive features built into VR headsets such as real-time captioning or hearing aid support; and movement disorders are another issue(Cipresso et al., 2018). While desktop computers and game consoles offer adaptive controllers, VR system compatibility with these controllers is often incomplete, complicating use. Notably, the adjustable game design meets the patient's needs and prevents abandoning difficult or simple actions, which not only improves adherence to the treatment plan and promotes early and sustained recovery, but also reduces the risk of dysfunction. Recent research advances have shown that the combination of VR technology and biofeedback not only improves immersion and realism, but also provides feedback that takes into account the user's experience and physiological limitations(Kourtesis et al., 2019). Upcoming improvements in the accuracy of biomonitoring devices continue to incorporate virtual environments where users make physical and mental adjustments based on feedback, thus providing a more immersive and personalized user experience.

## CRediT authorship contribution statement

**Zhuyue Ma:** Formal analysis, Conceptualization. **Li Sun:** Project administration, Methodology. **Yuanyuan Zhang:** Supervision, Funding acquisition.

## Ethics approval

Since this paper is a secondary citation and there is no direct investigative research on human biomedicine versus animals, ethical review is not applicable to this paper.

## Funding

This study was supported by the Advanced Training Programme for Key Talents of TCM Nursing in Jiangsu Province (Suzhou TCM Science and Education [2022] 18).

## Declaration of competing interest

The authors declare that they have no known competing financial interests or personal relationshaips that could have appeared to influence the work reported in this paper.

## Data Availability

The authors do not have permission to share data.

## Appendix A Supplementary data

Supplementary material Image 1
