# Supplementary material for: The role of virtual reality in breast cancer survivors: A scoping review
Source: Prev Med Rep. 2025 Mar 14;53:103032. doi: 10.1016/j.pmedr.2025.103032 (PMC12003002; doi:10.1016/j.pmedr.2025.103032)
Supplement: Supplementary file 1 — Supplementary material [file mmc1.docx]

Appendix 1: Preferred Reporting Items for Systematic Reviews and Meta-Analyses Extension for Scoping Reviews (PRISMA-ScR) Checklist.

| **SECTION** | **ITEM** | **PRISMA-ScR CHECKLIST ITEM** | **REPORTED ON PAGE #** |
| --- | --- | --- | --- |
| **TITLE** | | | |
| Title | 1 | Identify the report as a scoping review. | 1 |
| **ABSTRACT** | | | |
| Structured summary | 2 | Provide a structured summary that includes (as applicable): background, objectives, eligibility criteria, sources of evidence, charting methods, results, and conclusions that relate to the review questions and objectives. | 1-2 |
| **INTRODUCTION** | | | |
| Rationale | 3 | Describe the rationale for the review in the context of what is already known. Explain why the review questions/objectives lend themselves to a scoping review approach. | 4-5 |
| Objectives | 4 | Provide an explicit statement of the questions and objectives being addressed with reference to their key elements (e.g., population or participants, concepts, and context) or other relevant key elements used to conceptualize the review questions and/or objectives. | 6 |
| **METHODS** | | | |
| Protocol and registration | 5 | Indicate whether a review protocol exists; state if and where it can be accessed (e.g., a Web address); and if available, provide registration information, including the registration number. | NA |
| Eligibility criteria | 6 | Specify characteristics of the sources of evidence used as eligibility criteria (e.g., years considered, language, and publication status), and provide a rationale. | 7 |
| Information sources* | 7 | Describe all information sources in the search (e.g., databases with dates of coverage and contact with authors to identify additional sources), as well as the date the most recent search was executed. | 6-7 |
| Search | 8 | Present the full electronic search strategy for at least 1 database, including any limits used, such that it could be repeated. | 6-7 |
| Selection of sources of evidence† | 9 | State the process for selecting sources of evidence (i.e., screening and eligibility) included in the scoping review. | 6 |
| Data charting process‡ | 10 | Describe the methods of charting data from the included sources of evidence (e.g., calibrated forms or forms that have been tested by the team before their use, and whether data charting was done independently or in duplicate) and any processes for obtaining and confirming data from investigators. | 8 |
| Data items | 11 | List and define all variables for which data were sought and any assumptions and simplifications made. | 8 |
| Critical appraisal of individual sources of evidence§ | 12 | If done, provide a rationale for conducting a critical appraisal of included sources of evidence; describe the methods used and how this information was used in any data synthesis (if appropriate). | NA |
| Synthesis of results | 13 | Describe the methods of handling and summarizing the data that were charted. | 8 |
| **RESULTS** | | | |
| Selection of sources of evidence | 14 | Give numbers of sources of evidence screened, assessed for eligibility, and included in the review, with reasons for exclusions at each stage, ideally using a flow diagram. | 8-9 |
| Characteristics of sources of evidence | 15 | For each source of evidence, present characteristics for which data were charted and provide the citations. | 8-9 |
| Critical appraisal within sources of evidence | 16 | If done, present data on critical appraisal of included sources of evidence (see item 12). | NA |
| Results of individual sources of evidence | 17 | For each included source of evidence, present the relevant data that were charted that relate to the review questions and objectives. | Tables 1-3 |
| Synthesis of results | 18 | Summarize and/or present the charting results as they relate to the review questions and objectives. | 8-12 |
| **DISCUSSION** | | | |
| Summary of evidence | 19 | Summarize the main results (including an overview of concepts, themes, and types of evidence available), link to the review questions and objectives, and consider the relevance to key groups. | 12-13 |
| Limitations | 20 | Discuss the limitations of the scoping review process. | 15 |
| Conclusions | 21 | Provide a general interpretation of the results with respect to the review questions and objectives, as well as potential implications and/or next steps. | 16 |
| **FUNDING** | | | |
| Funding | 22 | Describe sources of funding for the included sources of evidence, as well as sources of funding for the scoping review. Describe the role of the funders of the scoping review. | NA |

Appendix 2: Indexed and Keyword Terms (Search Strategies) for Searching in Major Databases.

| **Database** | **Indexed and keyword terms** | **No. of articles retrieved** |
| --- | --- | --- |
| **PubMed** | ((((Virtual Reality[MeSH Terms]) OR (User-Computer Interface[MeSH Terms])) OR (Virtual Reality Exposure Therapy[MeSH Terms])) OR ((((((((((((((((VR[Title/Abstract]) OR (virtual realit*[Title/Abstract])) OR (virtual system*[Title/Abstract])) OR (virtual setting*[Title/Abstract])) OR (three-dimensional setting*[Title/Abstract])) OR (virtual environment*[Title/Abstract])) OR (three-dimensional environment*[Title/Abstract])) OR (computer simulat*[Title/Abstract])) OR (Gam*[Title/Abstract])) OR (google glass[Title/Abstract])) OR (google cardboard[Title/Abstract])) OR (head mounted display*[Title/Abstract])) OR (head mounted device*[Title/Abstract])) OR (HMD*[Title/Abstract])) OR (user-computer interface*[Title/Abstract])) OR (Virtual Reality Immersion Therapy[Title/Abstract]))) AND ((Breast Neoplasms[MeSH Terms]) OR ((((breast tumor*[Title/Abstract]) OR (breast cancer[Title/Abstract])) OR (breast carcinoma*[Title/Abstract])) OR (mammary gland carcinoma*[Title/Abstract]))) | 611 |
| **EMBASE** | #1 ‘breast neoplasms':ab,ti OR 'breast tumor*':ab,ti OR 'breast cancer':ab,ti OR 'breast carcinoma*':ab,ti OR 'mammary gland carcinoma*':ab,ti  #2 'virtual reality':ab,ti OR 'user-computer interface':ab,ti OR 'virtual reality exposure therapy':ab,ti OR 'vr':ab,ti OR virtual realit*':ab,ti OR 'virtual system*':ab,ti OR 'virtual setting*':ab,ti OR 'three-dimensional setting*':ab,ti OR 'virtual environment*':ab,ti OR 'three-dimensional environment*':ab,ti OR 'computer simulat*':ab,ti OR 'gam*':ab,ti OR 'google glass':ab,ti OR 'google cardboard':ab,ti OR 'head mounted display*':ab,ti OR 'head mounted device*':ab,ti OR 'hmd*':ab,ti OR 'user-computer interface*':ab,ti OR 'virtual reality immersion therapy':ab,ti  #3 #1 AND #2 | 4648 |
| **CINAHL** | S1 (MH “Virtual Reality”) OR (MH "User-Computer Interface") OR (MH "Virtual Reality Exposure Therapy")  S2 “VR” OR “virtual realit*” OR “virtual system*” OR “virtual setting*” OR “three-dimensional setting*” OR “virtual environment*” OR “three-dimensional environment*” OR “computer simulat*” OR “Gam*” OR “google glass” OR “google cardboard” OR “head mounted display*” OR “head mounted device*” OR “HMD*” OR “user-computer interface*” OR “Virtual Reality Immersion Therapy”.  S3 S1 OR S2  S4 (MH "Breast Neoplasms")  S5 “breast tumor*” OR “breast cancer” OR “breast carcinoma*” OR “mammary gland carcinoma*”  S6 S4 OR S5  S7 S3 AND S6 | 43 |
| **Web of Science** | Line 1: TS=(“Virtual Reality” OR “User-Computer Interface” OR “Virtual Reality Exposure Therapy” OR “VR” OR “virtual realit*” OR “virtual system*” OR “virtual setting*” OR “three-dimensional setting*” OR “virtual environment*” OR “three-dimensional environment*” OR “computer simulat*” OR “Gam*” OR “google glass” OR “google cardboard” OR “head mounted display*” OR “head mounted device*” OR “HMD*” OR “user-computer interface*” OR “Virtual Reality Immersion Therapy”)  Line 2: TS= (“Breast Neoplasms” OR “breast tumor*” OR “breast cancer” OR “breast carcinoma*” OR “mammary gland carcinoma*”)  Line 3: #1 AND #2 | 11,187 |
| **Cochrane Library** | #1 MeSH descriptor: [Artificial Intelligence] explode all trees  #2 MeSH descriptor: [Decision Making, Computer-Assisted] this term only.  #3 MeSH descriptor: [Decision Support Techniques] this term only  #4 MeSH descriptor: [Data Mining] this term only  #5 #1 OR #2 OR #3 OR #4  #6 “Artificial Intelligence" OR "Machine Learning" OR "Transfer Learning" OR "Deep Learning" OR "Fuzzy Logic" OR "Data Mining" OR "Decision-Making, Computer-Assisted"  #7 Expert NEXT System? OR Neural NEXT Network* OR Bayesian NEXT Network* OR Decision NEXT Support NEXT Technique? OR Computer NEXT Vision NEXT System?  #8 #6 OR #7  #9 #5 OR #8  #10 MeSH descriptor: [Nursing Care] this term only  #11 (Nurse? OR Staff NEXT Nurse?)  #12 ("Nursing Staff" OR "Nursing Personnel")  #13 #11 OR #12  #14 #9 AND #13 | 75 |
| **CNKI** | #1 虚拟现实 + 虚拟环境  #2 乳腺癌 + 乳腺肿瘤  #3 #1 * #2 | 15 |
| **Wanfang** | #1 虚拟现实 + 虚拟环境  #2 乳腺癌 + 乳腺肿瘤  #3 #1 * #2 | 19 |
| **CBM** | #1 虚拟现实 + 虚拟环境  #2 乳腺癌 + 乳腺肿瘤  #3 #1 * #2 | 8 |

Appendix 3: Data Extraction Form Used in this Scoping Review.

| **Author & year published:** | | | | | | **Country:** | | **Study setting:**  General wards  Outpatient clinics  Physiotherapy center  Laboratory  Community  Home  Not reported  Others: _____________ |
| --- | --- | --- | --- | --- | --- | --- | --- | --- |
| **Aim** |  | | | | | | | |
| **Study design** | | | True experimental  RCT  CCT  Quasi experimental  1 group post-test only  design  1 group pre-test post-  test design  Pre-test post-test  comparison only design  Post-test only  comparison group design | | | | Others  Crossover design  Qualitative study  Mixed study | |
| **Treatment Status** | | | after surgery  chemotherapy  radiotherapy  Others:  _____________ | | | | | |
| **Review Question 1: What does the scope of VR technology applied to BC care include?** | | | | | | | | |
| **Fields** | | | | Fatigue  Lymphoedema  Pain  Hair loss  Anxiety  Depression  Exercise fear  Limb function  Cognitive function  Muscle strength  Knowledge  Adherence to rehabilitation training  Others:  ___________ | | | | |
| **VR equipment** | | | | | VR Types  Immersive  Non-immersive  Specific equipment name  ______________ | | | |
| **Review Question 2: What are the feelings of VR technology application in BC patients?** | | | | | | | | |
| **BC patients' perception** | |  | | | | | | |
